# Adverse Drug Reactions in Hospital In-Patients: A Prospective Analysis of 3695 Patient-Episodes

**DOI:** 10.1371/journal.pone.0004439

**Published:** 2009-02-11

**Authors:** Emma C. Davies, Christopher F. Green, Stephen Taylor, Paula R. Williamson, David R. Mottram, Munir Pirmohamed

**Affiliations:** 1 The Royal Liverpool and Broadgreen University Hospitals Trust, Liverpool, United Kingdom; 2 School of Pharmacy and Chemistry, Liverpool John Moores University, Liverpool, United Kingdom; 3 Countess of Chester NHS Foundation Trust, Pharmacy: Martindale House, Countess of Chester Health Park, Chester, United Kingdom; 4 Centre for Medical Statistics and Health Evaluation, University of Liverpool, Liverpool, United Kingdom; 5 Department of Pharmacology and Therapeutics, The University of Liverpool, Liverpool, United Kingdom; Dr. Margarete Fischer-Bosch Institute of Clinical Pharmacology, Germany

## Abstract

Adverse drug reactions (ADRs) are a major cause of hospital admissions, but recent data on the incidence and clinical characteristics of ADRs which occur following hospital admission, are lacking. Patients admitted to twelve wards over a six-month period in 2005 were assessed for ADRs throughout their admission. Suspected ADRs were recorded and analysed for causality, severity and avoidability and whether they increased the length of stay. Multivariable analysis was undertaken to identify the risk factors for ADRs. The 5% significance level was used when assessing factors for inclusion in multivariable models. Out of the 3695 patient episodes assessed for ADRs, 545 (14.7%, 95% CI 13.6–15.9%) experienced one or more ADRs. Half of ADRs were definitely or possibly avoidable. The patients experiencing ADRs were more likely to be older, female, taking a larger number of medicines, and had a longer length of stay than those without ADRs. However, the only significant predictor of ADRs, from the multivariable analysis of a representative sample of patients, was the number of medicines taken by the patient with each additional medication multiplying the hazard of an ADR episode by 1.14 (95% CI 1.09, 1.20). ADRs directly increased length of stay in 147 (26.8%) patients. The drugs most frequently associated with ADRs were diuretics, opioid analgesics, and anticoagulants. In conclusion, approximately one in seven hospital in-patients experience an ADR, which is a significant cause of morbidity, increasing the length of stay of patients by an average of 0.25 days/patient admission episode. The overall burden of ADRs on hospitals is high, and effective intervention strategies are urgently needed to reduce this burden.

## Introduction

Adverse drug reactions (ADRs) in hospitalised patients can be divided into two broad categories: those that *cause* admission to hospital, and those that occur in in-patients *after* hospital admission. In a meta-analysis, using a random-effects model to reduce heterogeneity, Lazarou et al [1] showed that the total incidence of both categories of serious ADRs was 6.7%, of which 4.7% were responsible for admission and 2.1% occurred after admission, with an overall fatality rate of 0.32%. A recent Swedish study has also implicated ADRs as 7^th^ most common cause of death [2]. In a study of almost 19000 admissions, we were able to show that 6.5% of patient admissions to two National Health Service (NHS) hospitals in the UK were related to an ADR [3]. This incidence figure is broadly compatible with pooled data from older studies [1], [4], and with more recent studies [5], [6].

By contrast, data on ADRs occurring after hospital admissions are poor. Older studies have suggested that between 10–20% of patients suffer ADRs in hospital [7]–[10], while Lazarou *et al* suggested that 10.9% of patients suffer ADRs of all severities as in-patients [1]. A systematic review by Wiffen et al estimated that in the NHS in England, 1.6 million bed days, equivalent to 13.6 (400-bed) hospital equivalents annually are due to in-patient ADRs [4]. It is important to note that most of these data relate to studies that are decades old. With the changing demographics, the well-known predisposition of the elderly to ADRs, and the changes in medical practice that have occurred over the last few decades, there is a need for more data on the ADR burden in hospital in-patients.

As part of our overall strategy to determine the burden of ADRs in hospitals, after the completion of our ADR hospital admission study [3], we undertook a pilot study to establish the methodology for determining the burden of ADRs in in-patients. This pilot study of 125 in-patients showed that 19% of patients suffered ADRs, with patients experiencing an ADR spending 6.5 days longer in hospital than those without ADRs [11]. In this paper, we report the results of our large-scale prospective study which further explores the impact of ADRs on NHS hospital in-patients in terms of incidence, length of stay, costs involved, and factors that predispose patients to ADRs.

## Methods

### Patients and settings

The study was conducted on 12 wards (9 medical and 3 surgical) at the Royal Liverpool University Hospital (RLUH) over a six-month period between June and December 2005. The RLUH is a teaching hospital which serves a population of about 0.5 million with a total annual activity of 90,000 admissions. The study protocol was assessed and approved by the Liverpool Local Research Ethics Committee and the audit department at the RLUH, and the Research Ethics Committee at Liverpool John Moores University.

### Methods

For the purposes of this study, an ADR was defined according to the definition of Edwards and Aronson [12]. ADRs were identified on the basis that they were well recognised as evidenced by their inclusion in either the Summary of Product Characteristics [13] and/or the British National Formulary [14]. Only ADRs that occurred during admission as a result of drugs initiated or continued in hospital were included, while community acquired longstanding ADRs that were treated during the hospital stay were excluded (n = 17, 2.3% of all ADRs detected). ADRs that manifested no clinical signs, for example, suspected drug-induced abnormalities in blood test results were differentiated from those which caused clinical symptoms.

The study wards were a sample representative of the medical to surgical ward ratio in the hospital. Intensive care units and more specialist units such as the renal dialysis unit were excluded as our focus was on ADRs occurring in wards that are found in most UK general hospitals. The RLUH does not have psychiatric, paediatric or obstetrics and gynaecology wards, and thus our estimate of the incidence of ADRs excludes such patient groups. Patients admitted to the study wards during the data collection period were identified daily (Monday to Friday) by the research pharmacist using the hospital Patient Administration System (PAS). Patients whose admission did not include a weekday were therefore excluded, as were patients recorded on the PAS system following the daily check of ward lists, and discharged within one day prior to the next morning. Study wards were visited daily by the research pharmacist, and patients' drug charts, medical and nursing notes were reviewed for evidence of an ADR. Objective markers of ADRs, e.g. laboratory results, were identifiable from the patient notes and the hospital computer system, while subjective markers of ADRs, for example headache, nausea and rash were identified through patient notes, discussion with the ward team and, where appropriate, discussion with the affected patient. Clinical staff were informed that the study was taking place and could also refer directly either in person or through notification cards that were made available on the wards. The clinical ward pharmacists were consulted regularly regarding the possibility of ADRs on their designated wards. Following completion of the ward based data-collection period, retrospective case note analysis was performed to assess patient outcomes and to ensure that all available details regarding the ADR had been collected.

Suspected ADRs were classified in terms of causality [15] and avoidability [16] according to validated algorithms and assessed for suitability for Yellow Card Reporting to the Commission on Human Medicines and to the Medicines and Healthcare products Regulatory Agency (CHM/MHRA) [17]. ADRs were also classified as either type A (dose-dependent and predictable from the known pharmacology) or type B (idiosyncratic, no clear dose response relationship, and not predictable from the known pharmacology) according to the system introduced by Rawlins and Thompson in 1977 [18]. We chose this classification instead of the more recent DoTS classification [19] so that our data could be compared with previous studies. We also recorded severity of the ADRs according to the Hartwig severity scale [20], which was adapted from our pilot study [11], and subsequently modified to include two level 7 ADRs in order to differentiate between ADRs which directly, and those which indirectly, cause death.

Analysis for causality, avoidability, severity and suitability for yellow card reporting was done independently by investigators ED and CG. Overall there was approximately 60% agreement in the causality and avoidability assessments. Any discrepancies in scoring were then discussed before consensus was achieved between two investigators (ED and CG) in conjunction with the chief investigator (MP). Nine (1.2%) suspected ADRs were subsequently excluded by the investigating team, on the basis that an ADR was unlikely to be drug related according to the Naranjo algorithm [15]. The overall incidence of in-patient ADRs was defined as the total number of in-patient episodes which resulted in ADRs in relation to the total number of in-patient episodes in the study wards during the study period.

The length of stay for each patient was recorded using PAS data, enabling comparisons between patients with and without ADRs. Analysis of whether the ADR directly increased the length of stay, and the duration of this increase, was made following an assessment of the clinical features of the underlying disease and ADR, and after discussion with the ward team including the ward pharmacist and medical staff, and assessment of relevant case-notes. Clinical judgment was used to assess the additional length of stay attributable to the ADR. Thus, for example, if a patient had an ADR whilst waiting for nursing home placement, e.g. antibiotic-related *C. difficile* diarrhoea and the wait for placement independently exceeded the duration of the ADR, no additional length of stay was attributed to the reaction. Conversely, if a patient was ready for discharge, but an ADR occurred which required the patient to stay in hospital, the additional length of stay until recovery from the ADR was attributed to the reaction. All drugs including the causative drug(s) were recorded for all patients with ADRs. Given that there is no electronic system to capture information on medicines prescribed within our hospital, with the resources available, capturing information from all the patients would have led to incomplete and inaccurate information. We therefore decided to capture high quality data from a random sample of 1 in 10 in-patients on the same study wards.

ADRs which occurred despite specific prophylaxis against the ADR were recorded while the potential effect of polypharmacy on ADRs was measured by comparing the number of regular medicines taken by ADR patients on the first day of ADR with the number of regular medicines taken for the control sample (1 in 10 patients), assessed on the day of the in-patient stay where the patient received the maximum number of medicines. The most frequent ADRs relative to usage were calculated by using data of all drugs administered to one tenth of patients admitted. The frequency of the drug group causing a suspected ADR was divided by the number of times a drug in that class was administered in the control sample of patients (if≥1). The resulting ratio allowed drug groups to be further ranked by frequency of ADRs relative to drug use. The costs to the NHS were estimated using number of bed-days for additional length of stay based on the standard daily costs of NHS hospital episodes (£228) [21], consistent with the estimates used in our previous study [2].

### Statistical methods

The data were hierarchically structured, in that multiple ADR episodes can occur both within patients with multiple admissions to hospital and within a particular patient admission, hence the study collected data at the patient, admission, and ADR episode level. To compare ADR incidence between hospital wards, a generalised estimating equation (GEE) [22] model with compound symmetry was used to account for the within-patient correlation. This was considered more appropriate than a random effects model when there are small numbers of observations within patients [22].

For all other analyses, where a patient had multiple admissions or multiple ADRs, we used a patient's first ADR episode and analysed at the patient level only. The first ADR episode was used to simply assess the affected patient population and their risk factors (age, gender, number of medicines and placement on a medical or surgical ward) were identical or assumed to be broadly similar for patients who had multiple admissions. Binary outcomes were compared between groups using the chi-square statistic for assessing significance. Comparisons between groups with respect to continuous measures were made using the t-test or the Mann-Whitney U-test, depending on skewness, for assessment of statistical significance. The 5% level was used for assessing significance.

Risk factors for ADRs were identified by investigating the effects of age, gender, number of drugs prescribed and placement on a medical or surgical ward, on the time to ADR. Regression analysis was undertaken via the Cox proportional hazards model. Results are given in terms of the hazard ratio (HR) for a particular factor with accompanying 95% confidence interval (95%CI). The 5% significance level was used when assessing factors for model inclusion. The risk factor ‘number of regular drugs prescribed’ (calculated on day of admission where patient was taking most regular medications for control sample patients) had data available for 10% of the total sample (n = 374), and therefore was only investigated in this sample.

## Results

Over six months, there were a total of 3695 patient episodes assessed for ADRs involving 3322 patients. Out of these patient episodes, 545 (14.7%, 95% CI 13.6–15.9%) resulted in one or more ADRs. At the patient level, 524 of 3322 (15.8%) patients experienced at least one ADR. Women experienced significantly more ADRs (n = 308, 17.8%) than men (n = 216, 13.5%; χ^2^ = 11.6, df = 1, p<0.001). The median age was significantly higher in the ADR group at 72 years (Q1–Q3 56–81 years) compared with 61 years in the non-ADR group (Q1–Q3 41–77 years; U = 109, p<0.0001). More medical patient episodes (n = 406, 16.0%) than surgical episodes (n = 139, 12.0%) resulted in ADRs (χ^2^ = 10.12, df = 1, p<0.01). The incidence of ADR episodes varied further according to the specialty of the wards studied as shown in Table 1.The median length of stay for patient episodes that resulted in an ADR was 20 days (Q1–Q3 12–35 days), compared to 8 days (Q1–Q3 5–13 days; U = 138, p<0.0001) for those episodes without ADRs. Within the group of patients experiencing an ADR, the mortality was higher (n = 58, 10.7%), compared with 3.9% (n = 126) of patients who did not experience an ADR (χ^2^ = 42.4, df = 1, p<0.0001). ADRs contributed to 14 out of the 184 deaths (0.4% of patients admitted, 8.2% of all deaths), with one death (0.03% of patients admitted, 0.5% of all deaths) being directly attributable to the ADR, specifically GI bleed with diclofenac and dalteparin (see Table 2).

**Table 1:** Odds of experiencing an adverse drug reaction by ward type*.

Medical/Surgical Specialty†	Odds ratio (95%CI) in relation to breast/general surgical ward‡ (n = 555)	Number of patient episodes
Respiratory	3.65 (2.37 to 5.61)	298
Cardiology	3.34 (2.13 to 5.25)	256
Endocrine	3.19 (2.02 to 5.06)	242
Elderly medicine	3.06 (2.07 to 4.55)	544
Orthopaedic surgery	2.65 (1.81 to 3.90)	711
Rheumatology	2.55 (1.27 to 5.13)	76
Gastrointestinal/ Liver	2.43 (1.58 to 3.73)	390
Pharmacology	1.53 (0.95 to 2.47)	356
Infectious diseases	1.28 (0.75 to 2.20)	267

* Method used: General Estimating Equation (GEE) model with compound symmetry.

† One ward per specialty was assessed, with the exception of elderly medicine and orthopaedic surgery where two wards were assessed.

‡ Odds Ratios adjusted for multilevel structure.

**Table 2:** Deaths associated with adverse drug reactions.

Adverse drug reaction	No associated patient deaths	Drugs (number of deaths)	Avoidability (definite, possible, unavoidable)
Renal failure	7*	Gentamicin (1), bumetanide, valsartan (1), bumetanide, furosemide, spironolactone, ramipril (1), allopurinol, ceftriaxone, furosemide (1), diclofenac (1), furosemide, spironolactone (1), bumetanide, metolazone, perindopril, spironolactone, trimethoprim, potassium and calcium supplements (sando K, sandocal) (1, included hypercalcemia and hyperkalemia)	1 definite, 2 possible, 4 unavoidable
*Clostridium difficile* infection	5*	Ceftriaxone and ciprofloxacin and gentamicin (1), ceftriaxone, ciprofloxacin, lansoprazole (1), amoxicillin, cefuroxime, ciprofloxacin (plus lactulose and senna contributing to diarrhoea) (1), ceftriaxone, erythromycin, clarithromycin, co-amoxiclav (1), ceftriaxone, lansoprazole, trimethoprim (1)	3 possible, 2 unavoidable
GI Bleed	2	Dalteparin, diclofenac (1), aspirin, dalteparin, dipyridamole, enoxaparin (1)	1 definite, 1 possible
Ischaemic bowel	1	Glypressin (1)	1 possible

* In one patient both renal failure and *C.difficile* infection contributed to death.

A total of 733 ADRs were identified. Type A ADRs accounted for 690 (94.1%) of the ADRs while 232 (31.7%) ADRs fulfilled the requirements for reporting to the UK regulatory agency. Drug-drug interactions were linked to 433 (59.1%) of the ADRs; the majority of these were pharmacodynamic in origin (91.7%), for example, the use of multiple diuretics in patients with renal failure; 5.3% were pharmacokinetic, and 3% had a mixed pharmacokinetic-pharmacodynamic mechanism. The interactions will be described in more detail elsewhere.

The majority (n = 602, 82.1%) of the ADRs occurred as a result of initiation of the causative drug in hospital, of which 390 (65%) showed clinical signs. Similarly, of the 131 (62%) ADRs where the drug had been initiated prior to hospital admission, with the ADR occurring during admission, patients showed clinical signs in 81 (62%) cases. Tables 3 and 4 show detailed results of causality, severity and avoidability assessments.

**Table 3:** The adapted Hartwig severity scale and corresponding adverse drug reaction (ADR) frequency.

Severity Level	Description	Frequency of the ADR at each severity level; n (%)*
**1**	An ADR occurred but no change in treatment with suspected drug	**1 (0.1)**
**2**	The ADR required that required treatment with the suspected drug be held, discontinued, or otherwise changed. No antidote or other treatment required. No increase in length of stay	**151 (20.6)**
**3**	The ADR required that treatment with the suspected drug be held, discontinued, or otherwise changed, and/or an antidote or other treatment. No increase in length of stay	**413 (56.3)**
**4**	Any Level 3 ADR which increases length of stay by at least one day OR the ADR was the reason for admission	**152 (20.7)**
**5**	Any level 4 ADR which requires intensive medical care	**1 (0.1)**
**6**	The ADR caused permanent harm to the patient	**0 (0.0)**
**7a**	The ADR was indirectly linked to death of patient	**14 (1.9)**
**7b**	The ADR was directly linked to death of patient	**1 (0.1)**

* The denominator used was the total number of ADRs (n = 733).

**Table 4:** Causality and avoidability assessments of the adverse drug reactions in hospital in-patients.

Assessment	Category	Frequency of ADRs (n; %)*
Causality	Definite	23 (3.1)
	Probable	487 (66.5)
	Possible	223 (30.4
Avoidability	Definite	47 (6.4)
	Possible	344 (46.9)
	Unavoidable	342 (46.7)

* Denominator used was the total number of ADRs, n = 733.

In patient episodes associated with an ADR, the number of medicines taken was significantly higher (median 9, Q1–Q3 6–13) than in those episodes not associated with an ADR (median 6, Q1–Q3 4–10; U = 92644; p<0.0001). The drug groups most frequently implicated in the ADRs, and the frequency of their use in the study population are shown in Table 5. The most frequent causative drugs relative to usage were anticoagulants (warfarin), fibrinolytics (streptokinase) (4 ADRs), unfractionated heparin (3 ADRs), loop diuretics and allopurinol (5 ADRs).

**Table 5:** Drugs most frequently implicated in causing the adverse drug reactions (ADRs).

Drug group	No (%) ADRs	Top ten causative drug groups	Rank by frequency of use of drugs	Drugs (number of ADRs for each causative drug)	Adverse drug reactions
Loop diuretics	151 (20.6)	1	14	Furosemide (123), bumetanide (40)	Electrolyte disturbances, gout, hypotension, ileus, nausea, renal failure
Opioids	118 (16.1)	2	1	Morphine (88), tramadol (53), dihydrocodeine(10), fentanyl (8), codeine(8), oxycodone (7), pethidine (2)	Confusion, constipation, sedation, dizziness, respiratory depression, hallucinations, ileus, hypotension, itching, nausea, rash, dependence
Systemic corticosteroids	87 (11.9)	3	18	Prednisolone (67), dexamethasone (14), hydrocortisone (11), methylprednisolone (1), fludrocortisone (1)	Electrolyte disturbances, increased INR, bleeding, hallucination, hyperglycemia, fracture, hypertension, neutropenia, candidal infection
Beta-agonists (inhaled)	85 (11.4)	4	12	Salbutamol (85), terbutaline (4), salmeterol (3)	Electrolyte disturbances, nausea, tachycardia
Penicillins	66 (9.0)	5	6	Co-amoxiclav (34), amoxicillin (24), flucloxacillin (15), benzylpenicillin (7), penicillin v (1), ampicillin (1)	CDT, bleeding, rash, nausea, diarrhoea, increased INR, candidal infection
Oral anticoagulants	72 (9.8)	6	52	Warfarin (72)	Increased INR, bleeding
Cefalosporins	67 (9.1)	7	10	Ceftriaxone (40), cefuroxime (24), cefradine (3), cefaclor (2), Cefalexin (1), ceftazidime (1)	CDT, bleeding, increased INR, rash, nausea, neutropenia, candidal infection, worsening renal function
Compound analgesics (with opioid)	64 (8.7)	8	8	Co-codamol (58), co-dydramol (7)	Confusion, constipation, hypotension, sedation
Macrolide antibiotics	50 (6.8)	9	29	Erythromycin (34), clarithromycin (27)	CDT, bleeding, renal failure, deranged LFTs, diarrhoea, increased INR, rash, candidal infection, nausea
Low molecular weight heparins	50 (6.8)	10	6	Dalteparin (41), Enoxaparin (12)	Bleeding, heparin induced thrombocytopenia, electrolyte disturbances

Abbreviations: CDT – *Clostridium difficile* toxin disease; LFTs – liver function tests; INR – international normalised ratio.

ADRs occurred despite prophylaxis in 67 (9.1%) cases involving 10 types of ADR (constipation (35), electrolyte disturbances (10), renal failure (8), bleeding (5), raised INR (3), nausea (2), opioid withdrawal, opioid dependence, oral Candidal infection, and diarrhoea (all 1)). ADRs directly increased length of stay in 147 (27.0%) episodes, equating to 4.0% of all inpatient episodes and accounting for 934 out of 50145 (1.9%) bed days or 0.25 days/patient admission episode. For those episodes where length of stay was directly increased by an ADR, the median increase was 4 days (Q1–Q3, 2–7 days).

Table 6 gives results from multivariate risk factor analyses. The only significant predictor was the number of medicines (p<0.0001; HR 1.14; 95% CI 1.09, 1.20). Multivariable Cox regression confirmed these results, with the number of medicines as the only significant predictor. Therefore, on average, each additional medication multiplies the hazard of an ADR episode by 1.14. There may be a power issue in using the 10% sample, since the full dataset showed that both gender and age were significant risk factors for the ADR episode (p = 0.001 for both factors; HR (95% CI) 1.33 (1.12, 1.59) and 1.01 (1.0, 1.01) respectively). Comparing these with the 10% sample showed that the mean values from the full dataset were consistent with the 95% confidence intervals of the 10% sample. Furthermore, the 10% sample was representative of the whole sample set and unlikely to have introduced bias (Table 7).

**Table 6:** Risk factors for adverse drug reaction assessed by multivariate analysis.

Factor	N	Parameter Estimate	Standard Error	Chi-sq (df)	Pr>Chi Square	Hazard Ratio
Gender (F v M)	374	−0.026	0.24	0.012 (1)	0.9125	0.974
Ward Type (medical v surgical)	374	0.101	0.279	0.131 (1)	0.7178	1.106
Age	374	−0.002	0.007	0.060 (1)	0.807	0.998
Number of medicines	374	0.13	0.025	26.617 (1)	<0.0001	1.138

**Table 7:** Comparison of demographics of patients in 10% sample with the remainder of the study population.

Factor	10% Sample (n = 374)	90% Remainder of study population (n = 2948)
Gender (% Male)	49% (n = 185)	48% (n = 1410)
Ward Type (% medical)	69% (n = 258)	68% (n = 2001)
Age (years, (median, Q1–Q3))	62.5 (43–78)	63 (43–78)

## Discussion

This is the largest prospective study of adverse drug reactions in UK hospital in-patients. Our data suggest that at least 1 in 7 in-patient episodes is complicated by an adverse drug reaction. The incidence figure of 14.7% is consistent with our pilot study [11]. However, our figure is higher than the 3.5–7.3% incidence suggested in a systematic review [4]; this may be explained by the fact that pooling data from ADR studies with different designs can be problematical [23], [24] as illustrated by the widely differing estimates of ADR incidence determined in different studies in different populations (from 0.86% [25], to 37% [26]). In order to improve the accuracy of our assessments, individual causality assessments were undertaken using the Naranjo causality assessment tool [15]. There was 60% agreement in causality assessment and any disagreements were resolved by consensus discussions with a third assessor. Causality assessments are difficult, and inter-rater agreements vary enormously [27], but we feel that our methods were as robust as is possible in relation to assessment of individual cases. Thus, we feel that the prospective nature of this study, and the intensive nature of data collection and follow-up, similar in nature to two major recent studies of ADRs causing admission [3], [6] has resulted in, we believe, an accurate assessment of ADRs in adult hospital inpatients.

A clear limitation of our study is that it was conducted in one hospital and there is likely to be variation between different hospitals because of differences in the local population characteristics and the specialties within the hospitals. Nevertheless, we feel that the study population was selected from 12 wards which were representative of the clinical specialties (Table 1) commonly found in most acute hospitals and the age distribution of our study population was comparable to figures for all in-patient admissions in England [28]. Hospital episode statistics from the Department of Health in England state that in 2006–7 there were over 9 million adult admission episodes to NHS hospitals (excluding maternity admissions) [28]. There are 126,976 NHS beds in England, [29]. In our study, 1.9% of bed days were due to an ADR. Therefore we estimate that approximately 2000 bed days are due to an ADR at any one time, equivalent to approximately three 800-bed hospitals at 85% capacity. This figure, added to the estimate that the equivalent of seven 800-bed hospitals are filled with patients admitted with ADRs [3], suggests that ADR-related admissions and ADRs during hospitalization lead to the occupancy of ten 800-bed NHS hospitals. An accurate assessment of the financial cost of these ADRs is difficult, but a crude estimate based on an average cost of a bed day in the NHS in England suggests that the total costs are likely to exceed £171 million annually for ADRs occurring during admission. This is however likely to be an underestimate since the direct and indirect costs to patients such as loss of earnings due to extended stay or increased morbidity have not been measured, and neither were the costs which could be attributed to treating ADRs such as the prescribing of more medication and investigations, and involvement of clinical team external to the specialty to which the patient was admitted, all of which add to the overall ADR burden. As with our previous study [3], however, the figures provided here need to be interpreted cautiously as they represent an extrapolation from a single hospital to the NHS as a whole. However, taken together with our figure of £466 million for ADR-related admissions [3], we would estimate that ADRs cost the NHS in England in excess of £637 million annually, or approximately £5000 per hospital bed per year. This figure is comparable with adverse drug event (ADE) research from the United States ($8000 per hospital bed per year) [30], and with ADR research from mainland Europe (£4700 per hospital bed per year) [31].

### Implicated drugs and severity of reactions

The most frequently implicated drugs were opioid analgesics, diuretics, systemic corticosteroids, anticoagulants and antibiotics. This is in accordance with several other studies of hospital inpatients [32]–[34]. When adjusted for the frequency of prescription, warfarin, fibrinolytics and unfractionated heparin were the top three causes of ADRs. It is worrying to note that the same drugs, warfarin, loop diuretics and opioids, are being consistently implicated in different studies of ADRs; this may partly reflect their high usage, but nevertheless suggest that lessons have not been learnt from previous studies, and effective preventive strategies have not been put in place.

Approximately three quarters of adverse drug reactions were scored at level 3 or below on the Hartwig scale (Table 3) and by definition required intervention. These interventions ranged from stopping the causative medicine(s) to administration of specific antidotes, for example, naloxone for opioid-induced respiratory depression. The remaining ADRs were sufficiently serious to result in an increase in length of stay or admission to intensive care, and in some cases, death. The assessment of whether an ADR has increased the length of stay or caused death, and in particular whether it is due to the underlying disease or due to an ADR, can be extremely difficult; in our study, we undertook careful assessment involving the clinical team whenever possible, and taking into account individual patient factors such as the nature and severity of the underlying disease, and social factors which may have contributed to the length of stay more significantly than the ADR itself. Our estimate therefore is likely to have been relatively conservative.

### Prevention of adverse drug reactions

In this study, just over half of the ADRs were deemed possibly or definitely avoidable, which is consistent with the broad range of figures (30–70%) suggested in the literature [35], [36]. Given the considerable burden of ADRs, there is a need to put into place preventive strategies. Given the wide variety of drugs implicated, and the huge array of ADRs that we identified affecting almost every organ system in the body, prevention is likely to require complex multi-faceted intervention strategies. In our study, increasing age, admission to a medical ward, female gender, and number of regular medicines were identified as risk factors. Multivariable analysis of a representative sample of the data-set (Tables 6 and 7) showed that the only significant risk factor was the number of medicines the patient was taking, which may in itself be a reflection of age, gender and status as a medical patient. This is consistent with a number of previous studies [37]–[40]. Nevertheless, in order to confirm the results of our risk factor analysis it would be desirable to repeat the study in a second cohort. Given the increasing age of the population, and the trend towards polypharmacy, even in younger patients, the problem of ADRs is likely to remain a significant, if not increasing burden on our hospitals. Computerised prescribing and monitoring systems [41]–[43], the presence of pharmacists on ward rounds [44], [45], the need for better monitoring [46], and enhanced education of prescribing, leading to error reduction [47], are amongst the possible intervention strategies that have been suggested to be important in reducing the burden of ADRs. There is however a need for further research in this area, not only for the development of a robust evidence base to allow for prevention of ADRs, but also in the implementation of these strategies into hospital healthcare systems. Although it would be prudent to initially focus on the more serious ADRs, it is important to remember that even so-called non-serious ADRs, for example constipation from using opioids, can have a significant impact on the patient's quality of life, and also require the development of preventive strategies.

In conclusion, our study shows that ADRs are a significant problem in hospital inpatients, contributing to morbidity and mortality and resulting in considerable financial burden. Over half are definitely or potentially avoidable, and steps should be taken to introduce strategies to reduce their impact.
